# Association of inflammation and cognition in the elderly: A systematic review and meta-analysis

**DOI:** 10.3389/fnagi.2023.1069439

**Published:** 2023-02-06

**Authors:** Sofia Leonardo, Felipe Fregni

**Affiliations:** ^1^Ph.D. Department, Universidad Francisco Marroquín, Guatemala City, Guatemala; ^2^Center for Neuromodulation and Clinical Research Learning, Spaulding Rehabilitation Hospital and Massachusetts General Hospital, Boston, MA, United States

**Keywords:** elderly, inflammation, cytokines, CRP, IL-6, cognition

## Abstract

**Background:**

The development of mild cognitive impairment (MCI) and Alzheimer’s disease (AD) may be associated with an inflammatory process. Inflammatory cytokines may be a surrogate for systemic inflammation leading to worsening neurological function. We aim to investigate the association between cognitive impairment and inflammation by pooling and analyzing the data from previously published studies.

**Methods:**

We performed a systematic literature search on MEDLINE, PubMed, Embase, Web of Science, and Scopus for prospective longitudinal and cross-sectional studies evaluating the relationship between inflammation and cognitive functions.

**Results:**

A total of 79 articles were included in our systematic review and meta-analysis. Pooled estimates from cross-sectional studies have demonstrated an increased level of C-reactive protein (CRP) [Hedges’s g 0.35, 95% CI (0.16, 0.55), *p* < 0.05], IL-1β [0.94, 95% CI (−0.04, 1.92), *p* < 0.05], interleukin-6 (IL-6) [0.46, 95% CI (0.05, 0.88), *p* < 0.005], TNF alpha [0.22, 95% CI (−0.24, 0.68), *p* < 0.05], sTNFR-1 [0.74, 95% CI (0.46, 1.02), *p* < 0.05] in AD compared to controls. Similarly, higher levels of IL-1β [0.17, 95% CI (0.05, 0.28), *p* < 0.05], IL-6 [0.13, 95% CI (0.08, 0.18), *p* < 0.005], TNF alpha [0.28, 95% CI (0.07, 0.49), *p* < 0.05], sTNFR-1 [0.21, 95% CI (0.05, 0.48), *p* < 0.05] was also observed in MCI vs. control samples. The data from longitudinal studies suggested that levels of IL-6 significantly increased the risk of cognitive decline [OR = 1.34, 95% CI (1.13, 1.56)]. However, intermediate levels of IL-6 had no significant effect on the final clinical endpoint [OR = 1.06, 95% CI (0.8, 1.32)].

**Conclusion:**

The data from cross-sectional studies suggest a higher level of inflammatory cytokines in AD and MCI as compared to controls. Moreover, data from longitudinal studies suggest that the risk of cognitive deterioration may increase by high IL-6 levels. According to our analysis, CRP, antichymotrypsin (ACT), Albumin, and tumor necrosis factor (TNF) alpha may not be good surrogates for neurological degeneration over time.

## 1. Introduction

A variety of cell types can produce cytokines and non-antibody proteins. Interleukins (IL-1–24), tumor necrosis factors (TNFs), and transforming growth factors (TGFs Beta 1–3) are among the approximately 30 cytokines known. Cytokines are proteins that mediate cellular communication by autocrine, paracrine, or endocrine processes. Unique cytokine cell membrane receptors dictate the specificity of the cytokine response. The total response relies on its different components’ synergistic or antagonistic activities. Cytokine actions are the product of a complex network, frequently comprising feedback loops and cascades. The exact activities of different cytokines are hard to identify because of their pleiotropism (Papanicolaou et al., 1998; Wilson et al., 2002). IL-1, IL-6, and TNF are regarded as proinflammatory, but IL-4, IL-10, and IL-13 are typically considered anti-inflammatory in the periphery (Kronfol and Remick, 2000). The aging process is associated with a general decrease in immune function. Increasing age has been related to variations in blood levels of different cytokines (Wells et al., 2000). It has been extensively observed that serum IL-6 levels rise with aging in a variety of healthy groups (Wei et al., 1992; Ershler, 1993; Hager et al., 1994; Roubenoff et al., 1998). As a result, increases in IL-6 appear to be the normal outcome of aging, regardless of co-morbidity. Thymic atrophy and inhibition of thymopoiesis during aging may be linked to an increase in IL-6 with age (Sempowski et al., 2000).

It has been hypothesized that Alzheimer’s disease (AD) inflammation is linked and contributes to vascular dementia (Wilson et al., 2002). Central nervous system (CNS) levels of certain cytokines appear to increase as a function of age. Neurologically intact patients show a progressive increase in brain level of IL-1 and microglial activation with age (Sheng et al., 1998). Brain IL-6 levels in the mouse brain have been observed to rise with age, most likely as a result of increasing microglial output (Ye and Johnson, 1999). Cytokines’ impacts on cognition work in two ways, with systemic cytokines signaling the CNS inflammation and the behavioral effects of systemic cytokines producing systemic consequences that eventually negatively influence cognition. The cognitive manifestations of the abovementioned neural degeneration processes arise when acute and chronic excessive cytokine levels exceed a person’s homeostatic threshold, which may be measured by synaptic density or plasticity, and is termed a “cognitive reserve” (Wilson et al., 2002).

Beyond the conventional paradigms of cytokine-induced neurodegeneration and consequent cognitive impairment, processes affecting cognition in its broadest meaning must be investigated. The relationship between increased inflammatory markers in the serum and the occurrence and deterioration of cognitive degenerative diseases is still ambiguous. The correlation between inflammation and cognitive decline has been poorly studied. Older persons with no signs of dementia had higher levels of the inflammatory biomarkers interleukin-6 (IL-6) and C-reactive protein (CRP), according to a cross-sectional studies from the Netherlands (Schram et al., 2007) and the US (Yaffe et al., 2003). Consistently contradictory findings may be seen in longitudinal investigations of populations without dementia. Among older Finnish women, a higher CRP level at baseline was associated with worse cognitive performance 12 years later (Komulainen et al., 2007), while a cognitive decline was observed after 2 years in white and black elderly Americans (Yaffe et al., 2003). In white and black Americans, IL-6 predicted the cognitive decline after 2 years (Yaffe et al., 2003), while in Dutch older adults after 5 years (Weaver et al., 2002). Although, there are few systematic reviews and meta-analyses where authors have reviewed the level of biomarkers in cognitive decline (Swardfager et al., 2010; Holmes, 2013) but all of them have considered cross sectional studies only and mostly focused on AD vs. normal comparison in blood samples.

As a result, we aim to systematically review studies comparing cytokine levels between AD and mild cognitive impairment (MCI) vs. healthy controls from the cross-sectional as well as longitudinal studies to test the hypothesis of increased inflammatory levels associated with cognitive decline in this population.

## 2. Materials and methods

To conduct this systematic review and meta-analysis: we followed the Preferred Reporting Items for Systematic Reviews and Meta-Analyses (PRISMA) statements guidelines (Page et al., 2021), as well as the standards of the Cochrane Handbook for systematic review.

### 2.1. Literature search strategy

We searched the published literature in two electronic databases including MEDLINE, PubMed, Embase, and Web of Science and Scopus up to December 2021. Search terms were as follows:

(1) *Cognition OR cognitive decline OR cognitive function OR cognitive impairment OR cognitive loss OR memory.*
(2) Peripheral OR blood OR plasma OR plasm* OR serum OR sera.
(3) *Inflammatory markers OR inflammation OR cytokine OR chemokine OR IFN OR interleukin OR TGF OR TNF OR CRP.* Boolean operators (AND/OR) were used to combine the respective searches. We also manually searched the biography of the included studies for any additional relevant references cited within retrieved articles that were not retrieved during the literature search.

### 2.2. Eligibility criteria and study selection

Studies were included if they met the following criteria: (i) the study had a cross sectional or longitudinal prospective cohort design; (ii) the cross sectional studies should have reported data of AD, MCI with respect to controls, while in longitudinal studies, cognition performance was used at baseline and follow-up; in (iii) levels of cytokines were measured in blood; (iv) the longitudinal study measured the association of cytokine level and cognitive decline; (v) the article was available in English. Exclusion criteria included: (i) participants with dementia or cognitive impairment were included at baseline; (ii) the association between baseline cytokine level and cognitive decline was not reported; (iii) if the concentration of cytokine markers were measured in post-mortem samples. (iv) Small sample size less than 5 was used.

### 2.3. Quality assessment

The quality assessment was performed by two authors independently according to the Newcastle–Ottawa Scale, and disputes were resolved by discussion. The quality score was calculated based on three major components of cohort studies: quality of selection (zero to four stars), comparability (zero to two stars), and exposure and outcome of study participants (zero to three stars). A higher score represents better methodological quality. Studies were defined as high (greater than seven stars), medium (six to seven stars), or low quality (less than six stars).

### 2.4. Data extraction

Each type of dataset was extracted independently by two authors. Discrepancies were reconciled through full discussion and consensus among the reviewers. For longitudinal studies, the extracted data involved the following: (I) summary and baseline of patients included in our study including study ID (name of the author, year, and setting of the publication), study design, subjects at baseline, the proportion of females at baseline, mean age at baseline (years), mean follow up assessment of global cognition in months, and the conclusion of each study; (II) risk of bias (ROB) domains including three major components of cohort studies: quality of selection (zero to four stars), comparability (zero to two stars), and exposure and outcome of study participants (zero to three stars); and (III) the outcome measures. The outcome measures were extracted as odds ratio (OR) and 95% confidence intervals (CIs) for the adjusted model, and confounders were adjusted for in the regression analysis. For cross sectional studies, the sample sizes and mean (±SD) concentrations of markers were extracted and Hedge’s g was used for effect size (ES) for meta-analysis.

### 2.5. Data analysis

Statistical analyses were performed using Open Meta Analyst (*AHRQ, CEBM; Brown University, Providence, RI, USA*) and STATA version 16.0 (*StataCorp LLC, College Station, TX, USA*). We ultimately employed the random-effects model with the DerSimonian and Liard method (DerSimonian and Laird, 1986). From cross sectional studies, the sample sizes and mean (±SD) concentrations of markers were extracted and Hedges’s g was used for ES for meta-analysis. For longitudinal studies, all data were dichotomous (events and no events) and were pooled as weighted proportions and risk ratios (RRs) with 95% CI (Cumpston et al., 2019). Pooled rates of proportions were calculated through the Freeman–Tukey transformation meta-analysis of proportions using MedCalc (Version 15.0; MedCalc Software, Ostend, Belgium).

Heterogeneity between studies was examined visually and statistically through Chi-square and I2 tests: a *Q* statistic with *P* < 0.1 indicated heterogeneity, whereas I2 values of 0, 25, 50, and 75% represented no, low, moderate, and high heterogeneity, respectively (Higgins et al., 2003). When detecting considerable heterogeneity, we performed sensitivity analyses to ascertain the source of heterogeneity by excluding one study at a time in addition to and subgroup analyses. Publication bias was visually examined through funnel plot symmetry as well as mathematically through Egger’s regression test, Begg’s test, and Duval’s non-parametric trim-and-fill analysis (Begg and Mazumdar, 1994; Irwig et al., 1998; Duval and Tweedie, 2000).

## 3. Results

### 3.1. Search results and characteristics of included studies

**FIGURE 1:**
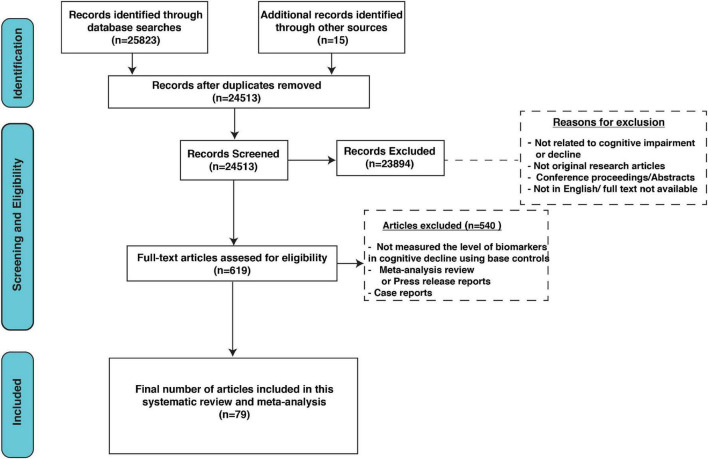
Preferred Reporting Items for Systematic Reviews and Meta-Analyses (PRISMA) flow diagram.

Our search extracted 25,513 unique citations after searching electronic databases. Following title and abstract screening, 619 full-text articles were retrieved and screened for eligibility. Of them, 540 articles were excluded, and 79 studies were reviewed in detail and included in this meta-analysis (PRISMA flow diagram; Figure 1).

The bibliography of the included randomized control trials (RCTs) was manually searched but added no further records. All studies were conducted between 2002 and 2021. Table 1 summarizes the characteristics of included patients and cross-sectional studies, while Table 2 represents longitudinal studies.

**TABLE 1:** Characteristics of included patients and cross-sectional studies.

Sr. No	References	Samples			Age (mean ± SD)			Female (%)			Diagnosis	Sample type	Assay type
		**AD**	**MCI**	**CI**	**AD**	**MCI**	**CT**	**AD**	**MCI**	**CT**			
1	Teunissen et al., 2003	34		61	73.0 ± 10.0		68.0 ± 6.7	59		43	DSM-IV; NINCDS-ADRDA	Serum	ELISA
2	Ciabattoni et al., 2007	44		44	73.0 ± 8.0		75.0 ± 7.0	56.8		61.4	NINCDS-ADRDA	Plasma	ELISA, Bio-Plex cytokine assay
3	Kassner et al., 2008	5		5	75.2 ± 3.5		71.0 ± 3.2	40		40	NINCDS-ADRDA	Serum	ELISA
4	Porcellini et al., 2008	195	69	830	79.2 ± 8.3	78.0 ± 8.0	73.0 ± 6.0	69.7	53.6	54.2	DSM-IV; NINCDS-ADRDA	Plasma	ELISA, Immunonephelometry
5	Davis et al., 2009	18		51	NA			NA			NINCDS-ADRDA; DSM-IVR	Serum	Immunoturbidimetric assay
6	Yarchoan et al., 2013	203	58	117	74.5 ± 7.7	71.6 ± 8.4	69.9 ± 10.1	58	52	68	NA	Plasma	ELISA
7	Bozluolcay et al., 2016	145		25	73.2 ± 10.2		73.1 ± 10.6	50		48	NINCDS-ADRDA	Serum	ELISA, Immunonephelometry
8	Villarreal et al., 2016	28	30	77	81.9 ± 9.2	81.2 ± 7.8	76.5 ± 6.7	78.6	66.7	64.9	NINCDS-ADRDA	Serum	Multiplex assay
9	Dukic et al., 2016	70	48	50	74.0 ± 7.6	72.0 ± 6.1	67.3 ± 7.6	61	65	60	NINCDS-ADRDA; the Petersen rating criteria	Serum	Complex
10	King et al., 2018	20	21	20	75.9 ± 6.7	78.5 ± 6.4	75.9 ± 7.3	25	66.7	20	NINCDS-ADRDA; NIA-AA	Plasma	Mixed
11	Choi et al., 2008	11		13	73.5 ± 4.0		68.5 ± 7.2	81.8		61.5	NINCDS-ADRDA	Serum, CSF	Immunoassay
12	Llano et al., 2012	15		7	70.2 ± 7.4		65.0 ± 5.2	20		28.6	NINCDS-ADRDA	Plasma	MSD
13	Leung et al., 2013	117	122	112	76.2 ± 6.1	73.9 ± 5.6	72.3 ± 6.7	66.7	49.2	53.6	MMSE; CDR; CERAD; ADAS-Cog	Plasma	Multiplex, ELISA
14	Rubio-Perez and Morillas-Ruiz, 2013	48		52	76.5 ± 3.5		79.0 ± 4.0	72.9		76.9	NINCDS-ADRDA	Serum	Immunoassay
15	Licastro et al., 2000	145		51	75.0 ± 12.0		78.0 ± 14.3	62.8		60.8	NINCDS-ADRDA; DSM-III R	Plasma	ELISA
16	De Luigi et al., 2002	58		47	NA			NA			NINCDS-ADRDA	Plasma	ELISA
17	Zuliani et al., 2007	60		42	78.5 ± 7.6		72.3 ± 5.8	65		46	NINCDS-ADRDA	Plasma	ELISA
18	Forlenza et al., 2009	58	74	21	76.3 ± 6.5	70.7 ± 10.3	69.9 ± 6.7	82.8	74.3	64.5	NINCDS-ADRDA	Serum	ELISA
19	Kamer et al., 2009	18		16	NA			78		94	DSM-IV; NINCDS-ADRDA	Plasma	Immunoassay
20	Richardson et al., 2013	24		35	79.0 ± 6.3		75.6 ± 7.6	63.8		61.8	NINCDS-ADRDA	Serum	ELISA
21	Bozluolcay et al., 2016	145		25	73.2 ± 10.2		73.1 ± 10.6	50		48	NINCDS-ADRDA	Serum	Immunoassay
22	Dursun et al., 2015	53	30	32	74.0 ± 3.9	74.4 ± 2.9	72.1 ± 3.4	NA			DSM-IV	Serum	ELISA
23	D’Anna et al., 2017	27		18	74.3 ± 8.0		70.7 ± 5.0	51.9		44.4	NINCDS-ADRDA	Serum	Pro human cytokine 10-Plex
24	Zhao et al., 2012	150		150	70.7 ± 4.3		69.9 ± 5.2	47.3		41.3	Peterson et al’s clinical criteria	Serum	ELISA
25	Wang et al., 2014	97	54	122	73.7 ± 9.4	76.6 ± 9.1	73.7 ± 8.4	44.3	57.4	54.0	DSM-IV; NINCDS-ADRDA	Plasma	ELISA
26	Villarreal et al., 2016	28	30	77	81.9 ± 9.2	81.2 ± 7.8	76.5 ± 6.7	78.6	66.7	64.9	NINCDS-ADRDA	Serum	Multiplex assay
27	Kim et al., 2017	35	29	28	77.7 ± 8.1	75.0 ± 7.5	72.0 ± 7.3	71.4	61.5	57.1	NINCDS-ADRDA; IWG criteria	Serum	ELISA
28	Zhu et al., 2017	96	140	79	77.3 ± 7.3	71.2 ± 8.1	68.3 ± 6.0	62.5	47.9	51.9	NINCDS-ADRDA	Serum	Luminex assays
29	Bonotis et al., 2008	49		21	75.0 ± 6.0		71.2 ± 4.4	57.1		52.4	INCDS-ADRDA	Blood	ELISA
30	Bozluolcay et al., 2016	145		25	73.2 ± 10.2		73.1 ± 10.6	50		48	NINCDS-ADRDA	Serum	ELISA, immunoassays
31	Eriksson et al., 2011	71		205	81.6 ± 5.3		81.3 ± 5.6	73.3		62	NINCDS-ADRDA; DSM-III R, IV	Serum	Immunoassays
32	Kamer et al., 2009	18		16	NA			78		94	DSM-IV; NINCDS-ADRDA	Plasma	Immunoassays
33	Kong et al., 2002	70		52	71.8 ± 9.7		69.0 ± 4.0	51.4		51.9	DSM-IV R, MMSE, ADL	Serum	ELISA
34	O’Bryant et al., 2016	79		65	76.1 ± 8.6		71.2 ± 9.2	70		68	NINCDS-ADRDA	Serum	Multiplex biomarker assay
35	Richartz et al., 2005	20		21	72.0 (60.0–88.0)	68.0 (59.0–82.0)		80	33	3	NINCDS-ADRDA	Serum	ELISA

**TABLE 2:** Characteristics of included patients and longitudinal studies.

References	Cohort	Setting	Subjects at baseline (*n*)	Female (%)	Mean age at baseline (years)	Mean follow up (years)	Conclusion
Dik et al., 2005	Longitudinal aging study Amsterdam	The Netherlands	1,284	51	75.4 ± 6.6	3	Serum inflammatory protein _1-antichymotrypsin (ACT) is associated with cognitive decline in older persons, whereas C-reactive protein (CRP), interleukin-6 (IL-6), and albumin are not.
Jordanova et al., 2007	N/A	Britain	290	57	65.5 ± 5.5	3	Raised IL-6 but not CRP predicted cognitive decline in this population inflammatory changes associated with cognitive decline may be specific to particular causal pathways.
Rafnsson et al., 2007	Edinburgh artery study	Britain	452	50	73.1 ± 5.0	4	Systemic markers of inflammation and hemostasis are associated with a progressive decline in general and specific cognitive abilities in older people, independent of major vascular comorbidity.
Schram et al., 2007 (Rotterdam study)	Rotterdam cohort	The Netherlands	3,874	58	72.1 ± 6.9	4.6	Systemic markers of inflammation are only moderately associated with cognitive function and decline and tend to be stronger in carriers of the APOE e4 allele. Systemic markers of inflammation are not suitable for risk stratification.
Schram et al., 2007 (Leiden 85-plus Study)	Leiden 85-Plus cohort	The Netherlands	491	65	85	5	Systemic markers of inflammation are only moderately associated with cognitive function and decline and tend to be stronger in carriers of the APOE e4 allele. Systemic markers of inflammation are not suitable for risk stratification.
Singh-Manoux et al., 2014	The Whitehall II Study	Britain	5,217	28	55.7 ± 6.0	5	Elevated IL-6 but not CRP in midlife predicts cognitive decline; the combined cross sectional and longitudinal effects over the 10-year observation period corresponded to an age effect of 3.9 years.
Weaver et al., 2002	The MacArthur study of successful aging	America	1,189	55	74.3 ± 2.7	7	There is a relationship between elevated baseline plasma IL-6 and risk for subsequent decline in cognitive function. These findings are consistent with the hypothesized relationship between brain inflammation, as measured here by elevated plasma IL-6, and neuropathologic disorders.
Yaffe et al., 2003	The health ABC study	America	3,031	52	73.6 ± 2.9	2	Serum markers of inflammation, especially IL-6 and CRP, are prospectively associated with cognitive decline in well-functioning elders. These findings support the hypothesis that inflammation contributes to cognitive decline in the elderly.
Alley et al., 2008	MacArthur study of successful aging	United States	533	51.8	74.4	7	Although high levels of inflammation are associated with incident cognitive impairment, these results do not generalize to the full range of cognitive changes, where the role of inflammation appears to be marginal.
Chen et al., 2014	Prospective, observational, cohort study	China	109	NA	74.1 ± 6.0	NA	Increased CRP was associated with cognitive impairment, and additive effects of increased CRP with hypertriglyceridemia and hyperglycemia on cognitive impairment were observed among elderly individuals.
Tilvis et al., 2004	Prospective cohort	Finland	650	73	75	5	Five-year decline was predicted by the presence of atrial fibrillation [relative risk (RR) 2.8], APOE4 (RR 2.4), elevated CRP (RR 2.3), diabetes mellitus (RR 2.2), and heart failure (RR 1.8). They also tended to increase 5-year all-cause mortality.
Adriaensen et al., 2014	Prospective, observational, cohort study	Belgium	303	37.3	84.3 ± 3.4	NA	Simple serum levels of IL-6 may be very useful in short-term identification or evaluation of global functional status in the oldest old.
Ashraf-Ganjouei et al., 2020	Longitudinal study	Iran	216	36.1	39.12 ± 20.19	NA	Healthy subjects with higher levels of CRP exhibit poorer performance in verbal learning memory and general wakefulness domains of cognition.
Baierle et al., 2015	Prospective cohort	Brazil	57	64.9	75	NA	Individuals with lower antioxidant status are more vulnerable to oxidative stress, which is associated with cognitive function, leading to reduced life quality and expectancy.
Beydoun et al., 2018	Prospective cohort	United States	2,574	55	46.9	4.64	Strong associations between systemic inflammation and longitudinal cognitive performance were detected, largely among older individuals (>50 y) and African-Americans. Randomized trials targeting inflammation are warranted.
Beydoun et al., 2019	Prospective cohort	United States	195	65	46.90 ± 1.00	4.64	Cytokines were shown to be associated with age-related cognitive decline among middle-aged and older urban adults in an age group and race-specific manner.
Boots et al., 2020	Longitudinal secondary analysis	United States	86	50	69.03 ± 6.65	NA	Peripheral inflammation is inversely associated with select cognitive domains and white matter integrity (but not WMHs), particularly in older Black adults. It is important to consider race when investigating inflammatory associates of brain and behavior.
Chi et al., 2017	Longitudinal study	United States	1,182	45	78.9 ± 3.4	8	Inflammation is associated with memory and psychomotor speed. In particular, systemic inflammation, vascular inflammation, and altered endothelial function may play roles in domain-specific cognitive decline of non-demented individuals.
Giudici et al., 2019	Prospective observational study	France	1,516	64.5	75.4 ± 4.5	5	Low-grade inflammation and hyperhomocysteinemia were both related with impairment on the combined IC levels among older adults after a 5-year follow-up. Identifying biomarkers that strongly associate with IC may help to settle strategies aiming to prevent the incidence and slow down the evolution of age-related functional decline and care dependency.
Goldstein et al., 2015	Longitudinal study	United States	278	63.7	57.4 ± 5.4	NA	There is strong association between IL-8 and cognitive performance in African Americans than Caucasians. This relationship should be further examined in larger samples that are followed over time.
Gunathilake et al., 2016	Longitudinal study	Australia	3,293	53	66.8 ± 7.8	NA	There is a weak positive association between obesity and cognitive performance in older persons, which is partially antagonized by inflammation and elevated fasting plasma glucose, but not hypertriglyceridemia.
Hajjar et al., 2018	Longitudinal cohort study	United States	511	68	49.1	4	Increased oxidative stress reflected by decreased glutathione was associated with a decline in executive function in a healthy population. In contrast, inflammation was not linked to cognitive decline. Oxidative stress may be an earlier biomarker that precedes the inflammatory phase of executive decline with aging.
Jung et al., 2019	Longitudinal cohort study	Republic of Korea	70	44.2	25.68 ± 3.89	NA	Cytokines, stress, and emotional and cognitive intelligence are closely connected one another related to brain structure and functions. Also, the pro-inflammatory cytokines tumor necrosis factor (TNF)-alpha and IL-6 had negative effects, whereas the anti-inflammatory cytokines [e.g., IL-10 and interferon (IFN)-gamma] showed beneficial effects, on stress levels, and multiple dimensions of emotional and cognitive intelligence.
Komulainen et al., 2007	Longitudinal cohort study	Finland	97	100	63.8	12	High serum hs-CRP concentration predicts poorer memory 12 years later in elderly women. Hs-CRP may be a useful biomarker to identify individuals at an increased risk for cognitive decline.
Marioni et al., 2009	Longitudinal cohort study	United Kingdom	3,350	72	61.9 ± 6.7	5	Increased circulating levels of CRP, fibrinogen, and elevated plasma viscosity predicted poorer subsequent cognitive ability and were associated with age-related cognitive decline in several domains, including general ability.
McHugh Power et al., 2019	Longitudinal cohort study	United Kingdom	116	66	65.81 ± 6.63	2	Inflammatory markers, cognitive function, social support, and psychosocial wellbeing were evaluated. A structural equation modelling approach was used to analyse the data. The model was a good fit (_108 2 = 256.13, *p* < 0.001).
Mooijaart et al., 2011	Prospective observational study	Scotland, Ireland, and the Netherlands	5,680	52	75.3	3.2	Plasma CRP concentrations associate with cognitive performance in part through pathways independent of cardiovascular disease. However, lifelong exposure to higher CRP levels does not associate with poorer cognitive performance in old age.
Sánchez-Rodríguez et al., 2006	Longitudinal cohort study	Mexico	189	75	66.8	NA	The elderly in urban areas have more oxidative stress and a higher risk of developing confidence interval (CI) compared with elderly individuals in a rural environment.
Sasayama et al., 2012	Longitudinal cohort study	Japan	576	75	45.1 ± 15.0	NA	Elevated IL-6 and soluble IL-6R levels in Ala carriers may have negative impact on acquiring verbal cognitive ability requiring long-term memory.
Sharma et al., 2016	Longitudinal case-cohort study	United States	1,298	45	79.0 ± 3.4	6	This study did not find strong evidence of the utility of the biomarkers evaluated for identifying individuals at risk of cognitive decline.
Shi et al., 2020	Prospective observational study	China	372	31.5	60.58 ± 7.86	2	IL-35 polymorphisms were not associated with cognitive decline in CHD patients over a 2-year period yet.
Sochocka et al., 2017	Longitudinal case-cohort study	Poland	128	64.8	55–90	NA	The comorbidity of the periodontal health status may deepen the cognitive impairment and neurodegenerative lesions and advance to dementia and Alzheimer’s disease (AD).
Yang et al., 2020	Longitudinal cohort study	China	122	49.18	NA	NA	T2DM patients have more cognitive impairment than T1DM patients. Changes in brain function connections and metabolites may be the structural basis of the differences in cognitive functional impairment. Inflammation is related to cognitive impairment in diabetes patients, especially in T2DM patients.
Yirmiya et al., 2020	Longitudinal cohort study	Israel	33	60.6	NA	NA	Sleep impairments in individuals with 22q11.2 deletion syndrome, which might negatively affect their cognitive functioning, and corroborate a potential role of immunological pathways in the 22q11.2 deletion syndrome neuro-phenotype.
Zheng et al., 2019	Case-control study	China	126	50	74.06	NA	Serum IL-6 and hs-CRP were associated with the risk of mild cognitive impairment (MCI) in Chinese patients with T2D. Serum folate might modify the association between serum hs-CRP and MCI in T2D patients.

### 3.2. The potential sources of bias

Following the Newcastle Ottawa Scale, the quality of the included studies ranged from moderate to high. The main concern was absent control groups. A summary of quality assessment domains with authors’ judgments is attached. Funnel plots of the inverse of the standard error vs. the ES demonstrated asymmetry. However, Egger’s test (*P* = 0.08) and Begg’s test (*P* = 0.13) indicated no small-study effects. Also, we employed the trim-and-fill approach to verify the robustness of the results, which exhibited no significant changes to the results when imputing three missing studies.

### 3.3. Comparisons between AD/control and MCI/control cross-sectional studies

**FIGURE 2:**
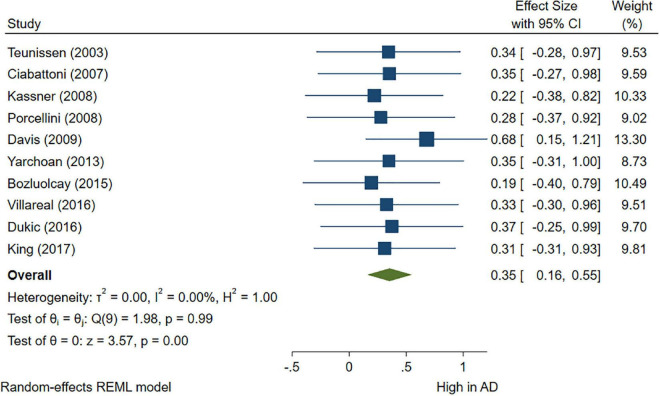
Forest plot of pooled Hedge’s g depicting high C-reactive protein (CRP) concentration in Alzheimer’s disease (AD) samples compared to controls.

**FIGURE 3:**
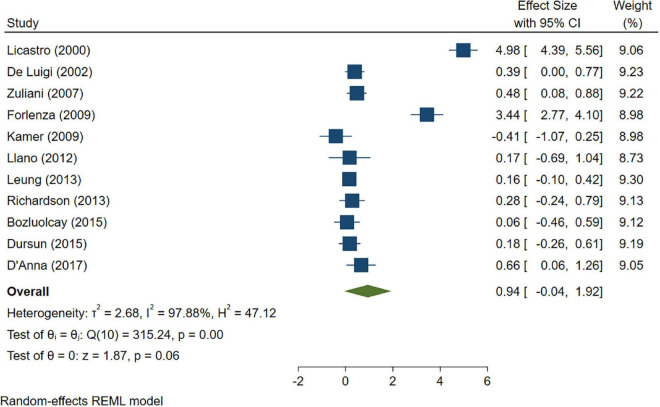
Forest plot of pooled Hedge’s g depicting high IL-1β concentration in Alzheimer’s disease (AD) samples compared to controls.

**FIGURE 4:**
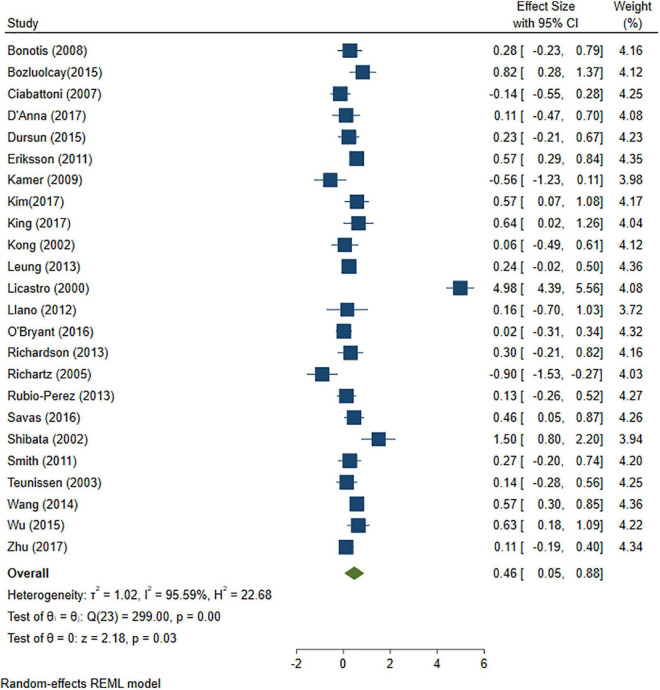
Forest plot of pooled Hedge’s g depicting high IL-6 concentration in Alzheimer’s disease (AD) samples compared to controls.

**FIGURE 5:**
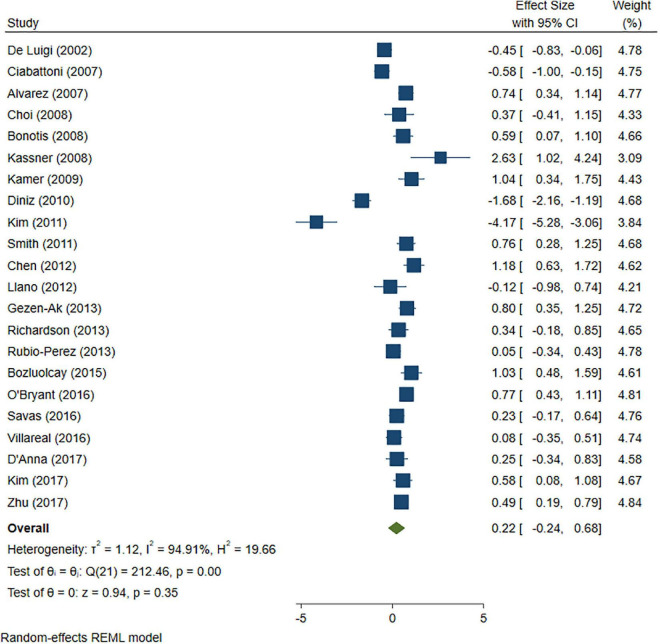
Forest plot of pooled Hedge’s g depicting high tumor necrosis factor (TNF)-alpha concentration in Alzheimer’s disease (AD) samples compared to controls.

**FIGURE 6:**
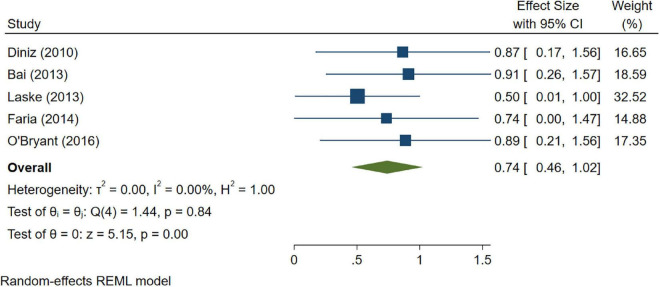
Forest plot of pooled Hedge’s g depicting high sTNFR-1 concentration in Alzheimer’s disease (AD) samples compared to controls.

**FIGURE 7:**
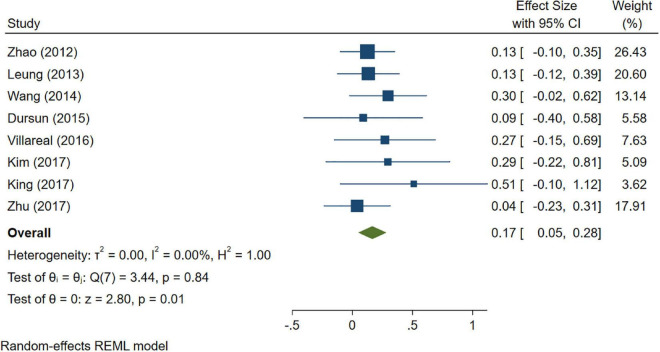
Forest plot of pooled Hedge’s g depicting high IL-1β concentration in mild cognitive impairment (MCI) samples compared to controls.

**FIGURE 8:**
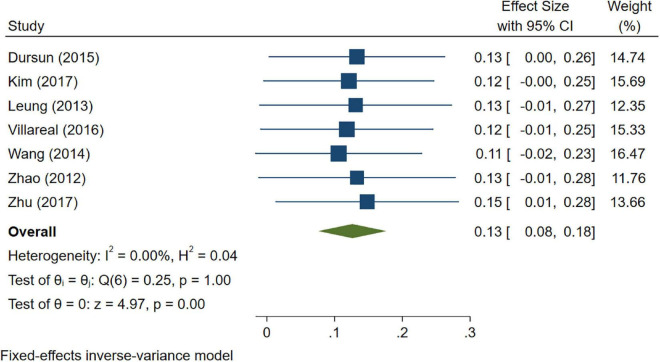
Forest plot of pooled Hedge’s g depicting high IL-6 concentration in mild cognitive impairment (MCI) samples compared to controls.

**FIGURE 9:**
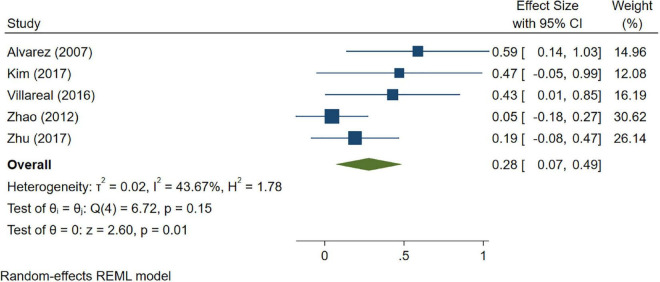
Forest plot of pooled Hedge’s g depicting high tumor necrosis factor (TNF)-alpha concentration in mild cognitive impairment (MCI) samples compared to controls.

**FIGURE 10:**
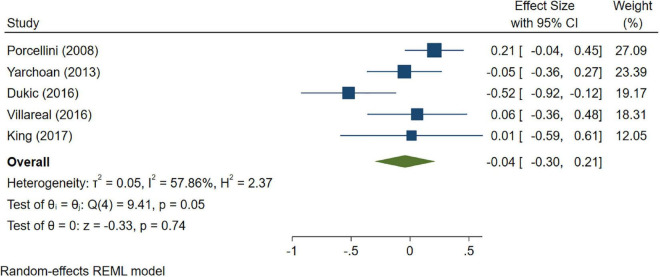
Forest plot of pooled Hedge’s g depicting high C-reactive protein (CRP) concentration in mild cognitive impairment (MCI) samples compared to controls.

A total of 46 studies were included in this analysis. Cross-sectional studies have demonstrated an increased level of CRP (Hedges’s g 0.35, *p* < 0.05) (Figure 2), IL-1β (0.94, *p* < 0.05) (Figure 3), IL-6 (0.46, *p* < 0.005) (Figure 4), TNF alpha (0.22, *p* < 0.05) (Figure 5), and sTNFR-1 (0.74, *p* < 0.05) (Figure 6) in AD compared to controls. Similarly, higher levels of IL-1β (0.17, *p* < 0.05) (Figure 7), IL-6 (0.13, *p* < 0.005) (Figure 8), TNF alpha (0.28, *p* < 0.05) (Figure 9), and CRP (0.21, *p* < 0.05) (Figure 10) was also observed in MCI vs. control samples.

### 3.4. Outcomes from longitudinal studies

#### 3.4.1. IL-6

**FIGURE 11:**
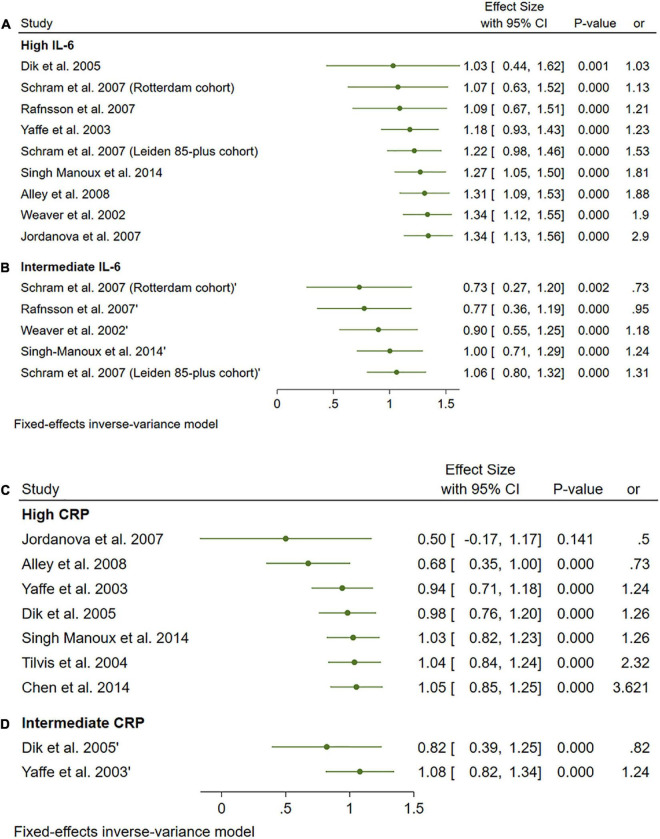
**(A)** Forest plot of pooled odds ratio (OR) for interleukin-6 (IL-6) high concentration and cognitive decline. **(B)** Forest plot of pooled odds ratio (OR) for IL-6 intermediate concentration and cognitive decline. **(C)** Forest plot of pooled odds ratio (OR) for C-reactive protein (CRP) high concentration and cognitive decline. **(D)** Forest plot of pooled odds ratio (OR) for CRP intermediate concentration and cognitive decline.

A total of 33 studies were included in this analysis and they followed subjects for an average of 58.35 months (min. 24 months and max. 144 months). High levels (>3.1 pg/ml) of IL-6 significantly increased the risk of cognitive decline [OR = 1.34, 95% CI (1.13, 1.56)]. However, intermediate levels (1.6–3.1 pg/ml) of IL-6 had no significant effect on the final endpoint [OR = 1.06, 95% CI (0.8, 1.32)]. The pooled analysis was homogeneous (I2 = 0%, *p* = 0.5) (Figures 11A, B).

#### 3.4.2. CRP

Interestingly, high and intermediate levels of CRP showed no significant impact on cognitive decline [OR = 1.08, 95% CI (0.74, 1.42)] and [OR = 1.05, 95% CI (0.64, 1.46)], respectively. The pooled analysis was moderately heterogeneous (I2 = 52.56%, *p* = 0.05), and heterogeneity did not resolve after further sensitivity analysis; thus, the random effect model was employed (Figures 11C, D).

#### 3.4.3. ACT, Albumin, and TNF

**FIGURE 12:**
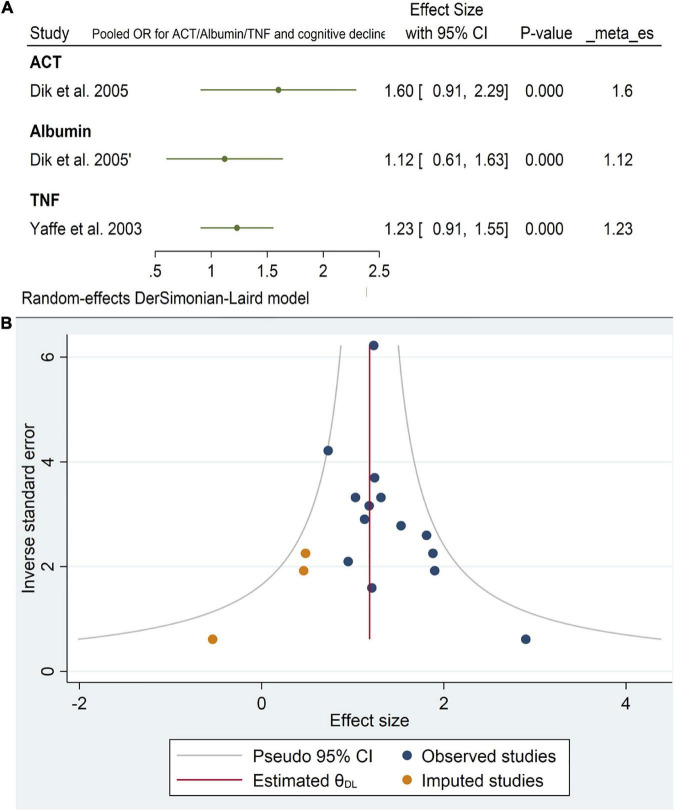
**(A)** Forest plot of pooled odds ratio (OR) for antichymotrypsin (ACT), albumin, tumor necrosis factor (TNF), and cognitive decline. **(B)** Funnel plot for the included longitudinal studies for interleukin-6 (IL-6) levels (intermediate and high).

Only two studies reported the effects of alpha1-antichymotrypsin (ACT), Albumin, and TNF alpha on cognitive outcomes. Neither ACT [OR = 1.6, 95% CI (0.9, 2.29)] Albumin [OR = 1.12, 95% CI (0.61, 1.63)] nor TNF [OR = 1.23, 95% CI (0.91, 1.55)] had a statistically significant effect on cognitive impairment. The pooled analysis was homogeneous (I2 = 0%, *p* = 0.5) (Figure 12A). Funnel plots of the inverse of the standard error vs. the ES demonstrated asymmetry (Figure 12B). However, Egger’s test (*P* = 0.08) and Begg’s test (*P* = 0.13) indicated no small-study effects.

## 4. Discussion

In this meta-analysis, we have compiled and analyzed the varied results from individual studies that looked at the relationship between AD and MCI and inflammatory markers in peripheral blood or cerebrospinal fluid. Different levels of inflammatory markers were found to be significantly different across the AD, MCI, and control groups. A total of 79 articles were included in our systematic review and meta-analysis which include cross-sectional and longitudinal cohort studies and case-control. Cross-sectional studies demonstrated several changes in the inflammatory marker levels in the comparisons between AD, MCI, and control groups.

Notably, IL-6 level was found to be significantly increased in patients with AD compared with controls and MCI vs. controls. This meta-analysis confirmed the elevated levels of interleukin family molecules including as IL-1, IL-6, and IL-8 in AD patients. These cytokines and chemokines connect with the existence and breakdown of amyloid-beta (Aβ) or tau proteins, which contribute to neurodegeneration pathways in AD (Teixeira et al., 2008). Specifically, IL-6 was revealed to have a potential characteristic that identifies the extent of cognitive decline in AD patients. Deposition of Aβ has been demonstrated to stimulate IL-6 production by microglia and astrocytes, which may speed the progression of AD’s degenerative cascade (Uslu et al., 2012). There are many common cytokines whose level were detected in AD and MCI conditions including CRP, IL1β, TNFα, IL6, and sTNFR1. However, their overall level was less in MCI compared to AD which suggest them as markers of neuroinflammation in AD and there may be an association between raised levels of cytokines in the blood and the development of AD.

### 4.1. Impact of systemic inflammation on cognitive performance

One hypothesis to explain the effects of increased cytokines in MCI and AD as compared to healthy controls is the impact of systemic inflammation on brain plasticity and function. Systemic inflammation raises pro-inflammatory cytokines such as IL-6, TNF-, and CRP, which may interact with the CNS in three ways: (1) pro-inflammatory cytokine transport proteins facilitate active trafficking across the blood brain barrier (BBB) for central activity (Dantzer et al., 2008; Fung et al., 2012). (2) Systemically generated cytokines may excite afferent nerves (e.g., the vagal nerve), which send inflammation to the brain stem. Vagal nerve projects to the solitary tract nucleus and higher brain areas (McCusker and Kelley, 2013). (3) Circulating cytokines reach outside-BBB organs. There, cells expressing toll-like receptors react to the increased inflammation by releasing pro-inflammatory cytokines, which may reach the brain by volume diffusion (Vitkovic et al., 2000b; McCusker and Kelley, 2013; Sankowski et al., 2015). When triggered peripherally, these three routes activate brain microglia and astrocytes to create pro-inflammatory cytokines, spreading the signal into the neuronal environment (Dantzer et al., 2008; Sankowski et al., 2015).

The blood-brain barrier, often known as the BBB, is an important component in both the preservation of the CNS’s highly specialized milieu and the facilitation of communication with the systemic compartment. Alterations to the BBB may be seen in a variety of CNS diseases, including AD. Some non-disruptive BBB alterations are mediated directly by cytokines (Ericsson et al., 1995). The cerebral endothelium expresses several cytokines, including IL-1, IL-6, and TNF- receptors. Activation of the endothelium is caused by IL-1 before that of neighboring brain regions, which suggests that activation of the BBB is an intermediary stage (Herkenham et al., 1998). It is interesting to note that IFN- decreases the transmigration of type 1 T helper lymphocytes without having any effect on the diffusibility of albumin. This suggests that the effect is not caused by a change in the tight junctions but rather by cytokine-induced non-disruptive changes that discourage neuroinflammation (Prat et al., 2005).

Systemic inflammation is also known to play an important role in altering the hormonal levels. There are a number of cytokines, including TNF-alpha, interleukin-1 beta (IL-1 beta), and IL-6 that work as feedback loops to inhibit the immunological response when the hypothalamic-pituitary-adrenal (HPA) axis is stimulated by the stress of trauma or exercise (Brenner et al., 1997; Licinio and Wong, 1997; Teblick et al., 2019). However, persistent increase of cytokines might potentially inhibit the HPA axis, which can lead to decreased levels of glucocorticoids, growth hormone, and adrenocorticotropic hormone (Teblick et al., 2019).

### 4.2. Increased cytokine levels as a marker of central nervous system inflammation

Preclinical and clinical research have demonstrated that infection-related peripheral inflammation contributes to the development and progression of CNS disorders such AD, personality disorders (PD), Multiple Sclerosis (MS), and stroke. Patients with AD have increased levels of Aβ in their brains due to peripheral inflammation (Le Page et al., 2018). In amyloid precursor protein (APP) transgenic mice, peripheral lipopolysaccharide (LPS) injection enhanced BBB permeability, enabling proinflammatory factors such as IL-6 and TNF- to infiltrate the brain and promote disease development (Jaeger et al., 2009; Takeda et al., 2013). The increased level of cytokines in included studies suggests that patients might have CNS inflammation.

### 4.3. Longitudinal studies and risk of IL-6

Results from longitudinal studies also showed high levels of IL-6 significantly increased the risk of cognitive decline. However, intermediate levels of IL-6 had no significant effect on the final endpoint. Likewise, neither CRP, ACT, Albumin, or TNF alpha showed a significant impact on cognitive decline.

The neuronal and glial cell function is modulated by a highly controlled network of cytokines and soluble cytokine receptors (Benveniste, 1998; Vitkovic et al., 2000a). This is related to their capacity to modulate neurotransmission. Increases in noradrenergic, dopaminergic, and serotonergic metabolism in the hypothalamus, hippocampus, and nucleus accumbens can be caused by both systemic and central cytokine administration (Mohankumar et al., 1991; Shintani et al., 1993; Linthorst et al., 1995; Merali et al., 1997). IFN- stimulates neuronal differentiation, while IL-1, IL-6, and TNF- have trophic effects on developing neurons and glia (Jonakait, 1997; Zhao and Schwartz, 1998). In the developing brain, IL-1 may also play a role in regulating the synaptic plasticity that underpins learning and memory (Zhao and Schwartz, 1998).

Jordanova et al. (2007), Singh-Manoux et al. (2014) reported that raised IL-6 but not CRP predicted cognitive decline in this population. Inflammatory changes associated with cognitive decline may be specific to particular causal pathways which is consistent with our findings. Weaver et al. (2002), Sasayama et al. (2012), Adriaensen et al. (2014) found a strong association between the level of IL-6 and short-term identification or evaluation of global functional status in the old. They reported that elevated IL-6 and soluble IL-6R levels in Ala carriers may have a negative impact on acquiring verbal cognitive ability requiring long-term memory. Mooijaart et al. (2011) found that plasma CRP concentrations associate with cognitive performance in part through pathways independent of cardiovascular disease. However, lifelong exposure to higher CRP levels does not associate with poorer cognitive performance in old age.

On the other hand, Yaffe et al. (2003), Chen et al. (2014) documented an association between CRP elevation and cognitive impairment which is inconsistent with our meta results. This may be because of the limited number of participants in these studies compared to our meta-analysis. Ashraf-Ganjouei et al. (2020) found that healthy subjects with higher levels of CRP exhibit poorer performance in verbal learning memory and general wakefulness domains of cognition.

Some other interleukins have a role in cognitive impairment other than IL-6. Goldstein et al. (2015) recorded strong relation between IL-8 and cognitive performance in African Americans than Caucasians. However, this relationship should be further examined in larger samples that are followed over time. Conversely, Shi et al. (2020) reported IL-35 polymorphisms were not associated with cognitive decline in coronary heart disease patients over 2 years.

Shen et al. (2019) reported that inflammatory marker levels were found to be significantly different in AD and control groups, supporting the idea that AD is accompanied by inflammatory responses in the peripheral and cerebro spinal fluid (CSF). In a previous review, Wilson et al. (2002) reported that cytokine-mediated inflammation in neurodegenerative disorders such as AD and vascular dementia is increasingly appreciated. Cytokines are an important part of stress activation in the hypothalamic-hypo physiologic-adrenal axis.

Our study is the first meta-analysis to our knowledge to differentiate between several inflammatory cytokines and their relation individually to cognitive impairment. In spite of the large sample size of our study and the strength of meta-analysis, these results are limited by the study design of included studies and the insufficient data about other inflammatory markers other than IL-6. Only two studies reported the effects of ACT, Albumin, and TNF alpha on cognitive outcomes. We need more controlled studies that have the least confounders to assure the strength of results.

### 4.4. Future perspectives: Inflammatory markers as a novel therapeutic target for AD

Although this meta-analysis only provides additional evidence for the association between some inflammatory markers and cognitive decline, it is worthwhile to discuss the potential impact for the development of novel treatments for AD. There has been an important debate on the current therapeutic target for AD, the amyloid β-protein. However, studies have not demonstrated that anti-amyloid therapies have induced significant therapeutic effects. In this context, some inflammatory markers, especially if targeted before the onset of cognitive deterioration, may be an interesting marker to target. For instance, it has been suggested that some diets, such as the Mediterranean-DASH Intervention for Neurodegenerative Delay (MIND) diets, can improve cognition and also have an anti-inflammatory effect (van den Brink et al., 2019). Also, non-invasive brain stimulation can also improve cognition (Martins et al., 2017) and reduce inflammation in animal models (Laste et al., 2012; de Oliveira et al., 2019; Toledo et al., 2021). One of the limitations of the current review is not including studies related to comorbid vascular dementia, which is connected with AD and MCI especially in elderly patients.

## 5. Conclusion

This review included inflammatory biomarkers in AD and MCI conditions supported from cross sectional and longitudinal studies. Cross sectional studies included in this review and meta-analysis demonstrated remarkable alterations in the peripheral levels of CRP, IL-1β, IL-6, sTNFR1, and TNF alpha. The findings from longitudinal studies indicated that the risk of cognitive deterioration has been substantially increased by high IL-6 levels. However, intermediate IL-6 levels did not affect the outcome significantly. Neither CRP, ACT, Albumin, or TNF alpha have had a major influence on cognitive degradation. These results provided further proof that AD and MCI are accompanied by inflammatory processes originating in the CNS.

## Data availability statement

The original contributions presented in this study are included in this article/supplementary material, further inquiries can be directed to the corresponding author.

## Author contributions

Both authors listed have made a substantial, direct, and intellectual contribution to the work, and approved it for publication.
